# HLA allele associations in idiopathic recurrent spontaneous abortion patients from India

**DOI:** 10.4103/0974-1208.39592

**Published:** 2008

**Authors:** Shankarkumar U, Pawar A, Gaonkar P, Parasannavar D, Salvi V, Ghosh K

**Affiliations:** HLA Department, Institute of Immunohaematology, 13^th^ Floor, KEM Hospital, Parel, Mumbai - 400 012, Maharashtra, India; 1 Department of Obstetrics and Gynaecology, KEM Hospital, Parel, Mumbai - 400 012, Maharashtra, India

**Keywords:** Cw*120201, DQB1*050301 association, HLA B*5701, India, RSA

## Abstract

**BACKGROUND:**

Rejection of semiallogenic foetus in recurrent spontaneous abortion (RSA) has been postulated to be a consequence of genetic and immunological phenomena.

**AIM:**

To evaluate the role of human leukocyte antigen (HLA) alleles in RSA in Indian couples.

**SETTINGS AND DESIGN:**

A case-control study.

**MATERIALS AND METHODS:**

Eighty-one randomly selected couples with unexplained three or more RSAs and a control group of 97 couples with live birth belonging to the same ethnic background, referred to the Gynaecology Department, KEM Hospital were included in the case-control study. Serological HLA A and B typing was done followed by molecular subtypes, defined using PCR-SSOP technique for HLA A, B, and C in 40 couples and DRB1* and DQB1* in 28 couples which were then compared with appropriate case 46 and 88 controls.

**RESULTS:**

Serologically A3 (15.43% *vs*. 4.43%; odds ratio (OR) = 4.34; *P* = 0.0002) and B17 (25.3% *vs*. 11.34%; OR = 3.49; *P* = 0.0001) were increased. Haplotype A1-B17 was significantly increased. Molecular subtyping revealed that A*030102 (11.25% *vs*. 4.34%; OR = 3.00; *P* = 0.07), B*5701 (11.25% *vs*. 1.08%; OR = 13.10; *P* = 0.003), Cw*120201 (25% *vs*. 4.34%; OR = 10.50; *P* = 2.05E-05), HLA DRB1*030101 (17.85% *vs*. 3.40%; OR = 7.6; *P* = 0.0001), DRB1*150101 (32.14% *vs*. 13.63%; OR = 4.8; *P* = 0.0003), and DQB1*060101 (35.71% *vs*. 29.34%; OR = 2.3; *P* = 0.004) were significantly increased in patients. A differential association was noticed when compared with reported world RSA patients.

**CONCLUSION:**

The HLA alleles A*030101, B*5701, Cw*120201, DRB1*030101, and DRB1*150101 as well as their associated ancestral haplotype may play a significant role in development of RSA in India.

Recurrent spontaneous abortion (RSA) is defined as a sequence of three or more consecutive spontaneous abortion. RSA is a heterogeneous condition, which may have many possible causes; more than one contributory factor is suggested to underlie the recurrent pregnancy losses. The major causes are attributed to genetic, endocrinological, or immunological. In the immunological cause, it may be autoimmunity or alloimmunity. In autoimmune, a woman may develop antiphospholipid antibodies, antithyroid antibodies, antinuclear antibodies, and antineutrophilic cytoplasmic antibodies. Alloimmune cases of RSA show involvement of human leukocyte antigen (HLA) and blood group antigens. The literature review reveals association of HLA alleles with RSA in various populations.[1–13] Strong association of HLA DR alleles have been shown with RSA.[4–9]

Numbers of studies indicate that there is a high sharing of HLA antigens in couples with RSA. In 1977, Komlos and his associates demonstrated the HLA allele sharing hypothesis concerning RSA.[14] Several studies have confirmed HLA antigens sharing between spouses in RSA.[12–13,15] Further meta-analysis of selected case-control studies suggested a slightly increased and significant risk of RSA among couples who shared at least one allele in HLA DR locus.[15] Other studies have also found nonsharing of HLA antigens.[16,17] In India, few studies have been conducted on HLA sharing, antipaternal cytotoixic antibodies, anticardiolipin antibodies, and lupus anticoagulant in RSA patients.[18,19] Studies from 120 Indian RSA women for nonclassical HLA G and HLA E associations have revealed that E*0101 and G*010103 were significantly associated for the first time.[20,21] Further, the role of 14 bp deletion in the HLA G gene toward the maintenance of pregnancy has shown no frequency difference among controls and RSA patients.[22]

Unexplained RSAs has many etiologies, many of them have immune dysfunctions which include the presence of cytotoxic antibodies, absence of maternal blocking antibodies, sharing of HLA antigens, and disturbances of killer cell immunoglobulin-like receptor dysfunction. The roles of Immunological and genetic characters have been implicated in RSA. Very little information from less frequent studies has been obtained in India. Therefore, the present study was conducted to evaluate the role of HLA in RSA.

## MATERIALS AND METHODS

A total of 81 couples with three or more RSAs and control group of 97 couples who had one or more children referred to the Gynaecology Department of KEM Hospital were included in the study. The RSA patients and the controls were in their reproductive age varying from 20 to 40 years. All the 81 couples were negative for conventional causes like hypertension, diabetes, infections due to toxoplasma, rubella, etc., hormonal dysfunction, thrombophilic risk factors, cytogenetics, autoimmune, and anatomical abnormalities. HLA serological typing and anti-HLA antibodies were done following the standard two-stage NIH microlymphocytotoxicity assay. In short from each individual peripheral blood was collected in heparin anticoagulant and the lymphocytes were separated on histopaque by density gradient centrifugation and the HLA A and B alleles were identified using specific HLA antisera of commercial and indigenous origin. Each specificity was defined by using three sera with good coefficient of correlation “*r*” value. The anti-HLA antibodies were investigated against the husband's lymphocytes. Further, molecular characterization of the HLA alleles were done by PCR-SSOP technique in 40 patients for HLA A, B, and C loci, in 28 patients for DRB1 and DQB1 loci which were then compared with appropriate 46 and 88 controls. The HLA antigen association between patients and normal controls were evaluated for allele frequency estimate, haplotype analysis, etiologic fraction, preventive fraction χ^2^-test, *P*-value, and odds ratio as described earlier.[23]

## RESULTS

Our analysis revealed that HLA A1, A3, and B17 were significantly increased while A9 decreased among the RSA patients from western India [Table 1]. Among the results of the 40 RSA patients characterized for their molecular A, B, and Cw subtypes compared with the normal controls. It was interesting to note that A*030101, B*5701, and Cw*120201 subtypes were significantly increased [Table 2]. Further, HLA DRB1 and DQB1 molecular typing among 28 RSA patients revealed that DRB1*030101, DRB1*150101, and DQB1*060101 were significantly increased while DRB1*010101, DRB1*110101, and DRB1*100101 were decreased among the RSA patients [Table 3]. Our comparative analysis from different world RSA patients revealed that our patients had differential association [Table 4]. Haplotype sharing has been significantly observed among 72 out of the 81 RSA couples (88.00%). Two locus haplotype analyses reveal that haplotype A1-B17 is significantly increased as well as haplotypes A10-B8, A3-B7, and A9-B35 were observed among RSA couples [Table 5]. The anti-HLA antibodies were found to be positive in 14.7% of the RSA women.

**Table 1:** Human leukocyte antigen distribution in RSA patients from Mumbai, India

HLA	Patients (*N* = 81)		Controls (*N* = 97)		OR	EF	PF	*P*-value
						
	*N*+	AF (%)	*N*+	AF (%)				
A1	40	24.69	32	16.49	1.981	0.24		0.026
A2	34	20.98	38	19.58	1.123			
A3	25	15.43	9	4.63	4.365	0.23		0.0002*
A9	20	12.34	40	20.61	0.467		0.21	0.02
A10	4	2.46	9	4.63	0.507			
A11	9	5.55	17	8.76	0.588			
A19	22	13.58	28	14.43	0.918			
A28	4	2.46	11	5.67	0.406			
B5	17	10.49	23	11.85	0.854			
B7	16	9.87	29	14.94	0.577			
B8	4	2.46	9	4.63	0.507			
B12	8	4.93	12	6.18	0.776			
B13	2	1.23	4	2.06	0.588			
B17	41	25.3	22	11.34	3.494	0.35		0.0001*
B22	7	4.32	7	3.6	1.216
B27	7	4.32	7	3.6	1.216			
B35	22	13.58	31	15.97	0.793			
B40	30	18.51	34	17.52	1.089			
B44	5	3.08	2	1.03	3.125	0.04		0.159

N+ - Number positive; AF (%)- allele frequency percentage; OR - Odds ratio; EF - Etiological fraction; PF-Preventive fraction

* Significant P-value

**Table 2:** HLA A, B, and C allele subtypes in Indian RSA patients

HLA	Patients (*N* = 40)		Controls (*N* = 46)		OR	χ^2^	EF	PF	*P*-value
						
	*N*+	AF (%)	*N*+	AF (%)				
A*0101	12	15.00	11	11.95	1.4	0.009			
A*02011	7	8.75	1	1.08	9.5	0.092	0.07		0.01
A*0211	8	10.00	12	13.04	0.7	0.001		0.03	
A*030101	9	11.25	4	4.34	3.0	0.004	0.14		0.07*
A*11011	5	6.25	13	14.13	0.4	0.026		0.03	0.07
A*2402	17	21.25	28	30.43	0.5	0.025		0.18	0.06*
A*2601	3	3.75	1	1.08	3.6	0.033	0.02		
A*31012	2	2.50	6	6.52	0.4	0.009		0.03	
A*3303	10	12.50	5	5.43	2.7	0.047	0.07		0.08
A*68011	7	8.75	2	2.17	4.7	0.064	0.06		0.04
B*0702	10	12.50	6	6.52	2.2	0.033	0.06		
B*0801	3	3.75	1	1.08	3.6	0.033	0.02		
B*1301	4	5.00	1	1.08	5.0	0.047	0.04		
B*1801	1	1.25	2	1.08	0.6	0.0002			
B*2705	2	2.50	1	1.08	2.4	0.02	0.01		
B*3501	5	6.25	1	1.08	6.4	0.061	0.04		0.06
B*3503	2	2.50	6	6.52	0.4	0.009		0.03	
B*3520	7	8.75	4	4.34	2.2	0.027	0.04		
B*3701	3	3.75	1	1.08	3.6	0.033	0.02		
B*4006	6	7.50	20	22.82	0.2	0.08		0.19	0.004*
B*4406	8	10.00	7	9.78	1.4	0.008	0.02		
B*4801	2	2.50	1	1.08	2.4	0.02	0.01		
B*510201	4	5.00	1	4.34	5.0	0.047	0.04		
B*52011	7	8.75	6	6.52	1.4	0.009	0.02		
B*5701	9	11.25	1	1.08	13.1	0.124	0.10		0.003*
Cw*0104	1	1.25	1	0.00	1.2	0.008			
Cw*02021	3	3.75	2	2.17	1.8	0.013	0.01		
Cw*03031	3	3.75	9	9.78	0.3	0.019		0.03	
Cw*04011	10	12.50	18	19.56	0.5	0.015		0.06	
Cw*0602	15	18.75	19	20.65	0.9	0.0002		0.03	
Cw*0603	1	1.25	0	0.00	46.0				
Cw*07011	6	7.50	4	4.34	1.9	0.018	0.03		
Cw*0702	12	15.00	8	8.69	2.0	0.054	0.07		
Cw*12021	20	25.00	4	4.34	10.5	0.236	0.16		2.05E-05†
Cw*15021	3	3.75	13	14.13	0.2	0.055		0.07	0.002*
Cw*1507	1	1.25	2	2.17	0.6	0.0002			
Cw*16041	1	1.25	1	1.08	1.2	0.008			

*N*+ - Number positive; AF (%) - allele frequency percentage; OR - Odds ratio; EF - Etiological fraction; PF - Preventive fraction

* Significant *P*-value

† Highly significant

**Table 3:** HLA DRB1 and DQB1 alleles in indian RSA patients

HLA	Patients (*N* = 28)		Controls (*N* = 88)		OR	χ^2^	EF	PF	*P*-value
						
	*N*+	AF (%)	*N*+	AF (%)				
DRB1									
*010101	1	1.78	15	8.52	0.2	0.019		0.04	0.071
*030101	10	17.85	6	3.40	7.6	0.15	0.14		0.0001*
*040301	1	1.78	7	3.97	0.4	0.001		0.01	
*070101	8	14.28	23	13.06	1.1	0.002			
*080101	1	1.78	1	0.56	3.2	0.024	0.02		
*090102	1	1.78	7	3.97	0.4	0.001		0.01	
*100101	7	12.50	15	8.52	1.6	0.012	0.04		0.071
*110101	1	1.78	19	10.79	0.1	0.031		0.06	0.027
*1404	4	7.14	9	5.11	1.5	0.007			
*150101	18	32.14	24	13.63	4.8	0.122	0.25		0.0003*
DQB1									
*0201	8	14.28	9	5.11	3.5	0.062	0.10		0.016
*030101	1	1.78	2	1.13	1.6	0.01			
*0302	3	5.35	3	1.7	3.4	0.034	0.03		
*030302	7	12.50	12	6.81	2.1	0.025	0.06		
*050101	10	17.85	13	7.38	3.2	0.063	0.11		0.015
*060101	20	35.71	52	29.34	2.3	0.042	0.19		0.004*

*N*+ - Number positive; χ^2^ - Chi square with Yates correction; AF (%) - allele frequency percentage; OR - Odds ratio; EF - Etiological fraction; PF - Preventive fraction

* Significant *P*-value

**Table 4:** A comparative distribution of HLA in RSA patients studied in the World

*Population*	Number	Studied	HLA alleles		Ref no.
					
	Patients	Controls	Associated	Protected	
Italy	47	65	B17		Vanoli *et al*.[11]
Italy			Cw5, Cw6 DR2		Sciorelli *et al*.[1]
UK			A2-B12		Johnson *et al*.[2]
Yugoslavia	35		A9		Gerencer *et al*.[3]
Italy			B44, DR5		Sbracia *et al*.[10]
Danish	234	360	DR1, DR3		Christiansen *et al*.[9]
Japan	27		DR4, DRB1*0405, 0406		Saski *et al*.[8]
Japan			DRB1*0403,04109	DQB1*0501	Hataya *et al*.[7]
Danish and	123		DR3	DR2	Christiansen *et al*.[6]
Czech					
Japan			DRB1*04		Takakuwa *et al*.[5]
Russia	130	57	A2, B18, B40		Vojvodic *et al*.[13]
Japan	89	207		B35	Imai *et al*.[12]
Japan	93	115	DRB1*1502		Takakuwa *et al*.[4]
India	81	97	A*030101, B*5701, Cw*120201	A*240201, Cw*15021‡	Present study
			DRB1*030101, DRB1*150101, DQB1*060101	B*4006m, DRB1*110101‡	Present study

**Table 5:** Significant haplotype associated in RSA patients in Mumbai

Haplotype	% HF	% LD	χ^2^	*T*-value
A1-B17	34.87	21.88	54.4	14.81
A10-B8	4.65	4.36	39.21	2.64
A3-B7	7.34	5.07	7.62	2.73
A9-B35	5.60	3.75	4.56	2.12
Haplotype sharing	72/81	-	88%	-

HF (%) - Haplotype frequency in percentage; LD (%) - Linkage disequilibrium in percentage; χ^2^ - Chi-square with Yates correction; *T*-value - Significant at >2

## DISCUSSION

The significant observations of the present study are (1) HLA A*030102, B*5701, Cw*12021, DRB1*030101, DRB1*150101, and DQB1*060101 are associated among the western Indian RSA women, (2) HLA A*240201, B*4006, Cw*15021, and DRB1*110101 are significantly reduced among the RSA women, and (3) ancestral Haplotype A1-B17 is significantly increased among the RSA women.

A comparative analysis of HLA in RSA patients reported from the world revealed a differential association among our patients. Earlier we had reported HLA A3 association in RhD immunised women from western India.[24] HLA B17 has been reported to be associated in Italian RSA patients.[11] HLA B17 has also been implicated in South Indian psoriasis patients.[25] Recently, haplotype A1-B17 has been reported to be involved in antineutrophil cytoplasmic antibodies (ANCA) production from western Indian population.[26] A significant reduced frequency of HLA B35 has been described in Japanese RSA patients.[12] Among Roman RSA couples, significant differences in HLA A2, B18, and B40 have been reported.[13] Increased frequencies of Cw5, Cw6, and DR2 in Italian RSA patients have been reported.[1] Further among the UK RSA patient population linkage disequilibrium between A2 and B12 has been reported.[2] Various studies on HLA DR and DP loci have shown associations with RSA in Italian,[10] Danish,[6,9] and Japanese[5,7,8] populations. Recently, HLA DRB1*1502 has been reported to be significantly associated in Japanese RSA patients.[4] The reports on antigen sharing in couples with recurrent miscarriage led to several studies assessing the role of HLA antigens and their influence on the outcome of pregnancy.[27] In fact, couples sharing at HLA A, HLA B, HLA C, HLA DR, and HLA DQ loci has been reported to be positively associated with the risk of RSA.[28] Since 1977, increased HLA sharing among spouses has been associated with RSAs; later more specific HLA DR and/or DQ antigens were suggested. HLA sharing has also been reported in couples that fail to achieve pregnancy with multiple cycles of assisted reproductive techniques. Studies examining the association of HLA sharing with a risk of RSA have yielded inconsistent results, in terms of whether or not couple sharing is significantly related to the outcome and which particular HLA gene may be responsible. Meta-analysis of selected case-control studies suggested a significant risk of RSA among couples who shared at least one allele at the HLA-DR locus, but not at the other HLA loci.[14] The discrepant results in the HLA and RSA studies were attributed to inconsistent definitions of recurrent miscarriage and of the control groups, the small number of samples, and the different tissue typing methods used to investigate the HLA loci. Therefore, we had done serology initially and then molecular characterization for the allele subtypes in this case-control study to define all the HLA loci.

Due to variation in immunization and the resultant outcome, a wide variation in the incidence of anti-HLA antibodies in the sera of pregnant women has been reported in the literature. The values ranged from 7.3 to 36%.[24] It has been reported earlier that 27.8% of RSA women had anti-HLA antibodies from northern India.[18] In our study, we have found an incidence of 14.7% anti-HLA antibodies, which suggests that the blocking factors as well as immunity toward the husband's lymphocytes could be different among western Indian RSA couples.

Recently, the involvement of killer cell immunoglobulin-like receptors (KIR) in abortion with specific HLA molecules on trophoblast (HLA C, G, and E) may influence the outcome of pregnancy has been suggested.[29] Studies conducted in reproductive immunology component of the 14th International Histocompatibility Workshop has suggested that women with alloimmune abortions or implantation failures may possess a rather uncommon KIR repertoire characterized by increased presence of activating receptors and may have a higher potential decidual natural killer (NK) cell activation, thus increased risk for an adverse reproductive outcome.[30] It could be possible that HLA Cw*12021, the associated allele along with its associated ancestral haplotypes A3-B17-CwBk-DR3 in India could have an immunopathological role via the HLA C-KIR related activation which leads to an adverse reproductive outcome. This remains to be still elucidated though we have proved that ancestral haplotype A1-B17-Cw6-DR7 has been associated with ANCA production in renal-associated vasculitis from India. Further we have provided evidence of Cw*1507 allele to be strongly associated in HIV-1 infected Indian patients (personnel communication). Future studies involving these parameters along with the other immunological evaluations are necessary to substantiate the immunological basis of rejection in RSA.
